# Concordance Between Common Hypertension Control Algorithms in Electronic Medical Record Data

**DOI:** 10.5888/pcd14.170032

**Published:** 2017-09-14

**Authors:** Victoria M. Nielsen, Amy Bettano, Mark Josephson, Laura Nasuti, W.W. Sanouri Ursprung

**Affiliations:** 1 Massachusetts Department of Public Health, Boston, Massachusetts; 2 Massachusetts League of Community Health Centers, Boston, Massachusetts

## Abstract

Because quality improvement metrics and treatment guidelines are used to conduct research, evaluate care quality, and assess population health, they should, ideally, align. We used electronic medical record data to analyze variation between blood pressure control estimates calculated by using thresholds derived from National Quality Forum 0018 (NQF 0018) and Joint National Committee (JNC) treatment guidelines in a cohort of patients with hypertension. Percentage of patients with controlled blood pressure derived from each quality improvement or treatment guideline cutoff varied up to 16.1 percentage points. This variance demonstrates that discrepancies in blood pressure thresholds produce considerable variation in estimates; thus, treatment guidance and metrics should be selected carefully.

## Objective

Hypertension affects 1 in 3 Americans and results in adverse outcomes and significant health care expenditures (1,2). Although algorithms used for quality improvement and treatment guidelines in hypertension care use different blood pressure (BP) thresholds, no previous analyses have explored how much variation exists when these differing thresholds are used to estimate BP control in the same population (3–5). It is important to quantify the variation that exists when different BP thresholds derived from clinical quality metrics and treatment guidelines are used to estimate BP control, especially when the measures and guidelines do not align. By using such information, guidance can be provided to clinicians on how to better integrate both treatment guidelines and clinical quality metrics into clinical practice. In addition, attempts to synthesize published research that uses different BP cutoffs to estimate BP control may result in findings that are not comparable. Consideration of this variation is critical in interpreting and generalizing results that apply different BP control algorithms to clinical data.

## Methods

By using electronic medical record (EMR) data, concordance was assessed between 3 BP control algorithms. These algorithms applied BP thresholds derived from 2 treatment guidelines (Joint National Committee) and one clinical quality metric (National Quality Forum 0018) used in hypertension care (3–5). The following 3 BP control algorithms were defined on the basis of BP thresholds derived from each guideline or metric:

1. The Seventh Report of the Joint National Committee on Prevention, Detection, Evaluation, and Treatment of High Blood Pressure (JNC7): Patients without diabetes or chronic kidney disease: systolic BP <140 mm Hg, diastolic BP <90 mm Hg; patients with diabetes or chronic kidney disease: systolic BP <130 mm Hg, diastolic BP <80 mm Hg (3).
2. 2014 Evidence-Based Guideline for the Management of High Blood Pressure in Adults: Report From the Panel Members Appointed to the Eighth Joint National Committee (proposed revisions for JNC7, or colloquially known as JNC8): Patients aged <60 y, who have diabetes, or who have chronic kidney disease: systolic BP <140 mm Hg, diastolic BP <90 mm Hg; patients aged 60 y or older: systolic BP <150 mm Hg, diastolic BP <90 mm Hg (4).
3. Controlling High Blood Pressure, National Quality Forum 0018 (NQF 0018): Systolic BP <140 mm Hg, diastolic BP <90 mm Hg (5).

We analyzed encounter-level EMR data, including demographics, diagnoses, and vital signs from 19 community health centers. Data spanned approximately 3.5 years (September 2013–March 2017). Because data were collected by the Massachusetts Department of Public Health for quality improvement, review by an institutional review board was not required.

A cohort of patients with primary hypertension (*International Classification of Diseases, 9th Revision, Clinical Modification* [ICD-9-CM] and *International Classification of Diseases, 10th Revision, Clinical Modification* [ICD-10-CM], codes 401.*/I10) was assembled. Primary hypertension was selected because clinical priorities may be different when hypertension is secondary to other conditions. Patients were included in the cohort if they had a diagnosis of primary hypertension any time prior or up to 6 months into the measurement period. Patients could meet this criteria via 1 of 2 pathways: 1) a pre-existing hypertension diagnosis, ascertained by presence of an ICD code in a historical diagnosis file (ie, a separate data set that solely contains data on pre-existing conditions), or a diagnosis of hypertension at any visit during the 2.5 years of data preceding the 1-year measurement period; or 2) patients newly diagnosed with hypertension who had a diagnosis at any visit in the first 6 months of the measurement period. In addition, during the 1-year measurement period, patients had to be aged 18 to 85 years, could not be pregnant, and had to have had at least 1 visit with a health care provider (the BP data recorded at this visit was used to estimate BP control). The algorithm used to construct the cohort is similar to that used for NQF 0018 but with a few deviations (5). First, patients with chronic kidney disease were not excluded, because this condition had key strata in the BP control algorithms. In addition, NQF specifications include only patients diagnosed in the first 6 months of the measurement period. However, in this analysis, patients were also included if they had a diagnosis any time before the measurement period, a change intended to maximize the size and robustness of the patient cohort.

To estimate BP control, the measurement period consisted of the final 1 year of data. Patients’ BP control was determined by using measurements at last office visit; if patients had a visit but no measurements were documented, they were deemed not in control. Finally, if diagnosis of primary hypertension occurred only in the first 6 months of the measurement period, the patient must have had an additional visit in the second half of the measurement period to calculate BP control; otherwise, the patient was excluded from the cohort.

## Results

By using the ICD-9-CM and ICD-10-CM diagnosis codes for primary hypertension, a cohort of 44,780 patients was assembled and percentage of BP control calculated. The average patient age was 58.1 years (Table 1), and approximately half of the sample was male (20,398 patients, or 45.6%). Roughly one-third of patients (n = 15,779 [35.2%]) were non-Hispanic white, 23.2% (n = 10,369) were non-Hispanic black, and 28.9% (n = 12,950) were Hispanic (Table 1).

**Table 1:** Characteristics of Patients (N = 44,780) With Hypertension, Massachusetts, September 2013–March 2017^a^

Characteristic	Measure
**Age, y**	58.1 (12.6)
**Blood pressure^b^**	
Systolic, mm Hg	131.9 (16.5)
Diastolic, mm Hg	79.3 (10.4)
**Sex**	
Male	45.6 (20,398)
Female	54.4 (24,378)
**Race/ethnicity**	
Non-Hispanic white	35.2 (15,779)
Non-Hispanic black	23.2 (10,369)
Hispanic	28.9 (12,950)
Other	12.7 (5,682)
**Key algorithm substrata**	
Patients with diabetes or chronic kidney disease	36.6 (16,408)
Patients ≥60 y	47.2 (21,157)

a Based on encounter-level electronic medical record data from 19 community health centers. Values are % (No.) unless otherwise indicated and may not sum to 100% because of rounding or missing data.

b Blood pressure at last visit where blood pressure was recorded.

Point estimates varied widely by the 3 algorithms. Within the same cohort, NQF 0018 returned a control percentage of 71.7%, whereas JNC7 resulted in 61.3%, and proposed revisions to JNC7 resulted in 77.4% (Table 2). In reviewing the substrata of each hypertension control algorithm, percentage of blood pressure control exhibited noteworthy patterns: JNC7 had the lowest percentage of control among patients with diabetes or chronic kidney disease (43.9%), while proposed revisions to JNC7 demonstrated a markedly higher rate of control in patients aged 60 years or older (83.5%).

**Table 2:** Hypertension Control, by 3 Algorithms, Overall and by Key Algorithm Substrata, Patients (N = 44,780) With Hypertension, Massachusetts, September 2013–March 2017^a^

Characteristic	JNC7	JNC8^b^	NQF 0018
Overall	**61.3**	**77.4**	**71.7**
**Age, y**			
<60	62.8	71.9	71.9
≥60	59.6	83.5	71.4
**Comorbid diabetes or chronic kidney disease**			
Patients with diabetes or chronic kidney disease	43.9	79.4	72.3
Patients without diabetes or chronic kidney disease	71.4	76.2	71.4
**Sex**			
Male	59.7	75.5	70.5
Female	62.6	78.9	72.7
**Race/ethnicity**			
Non-Hispanic white	64.3	79.0	73.2
Non-Hispanic black	57.6	73.7	68.0
Hispanic	61.6	78.4	73.4
Other	59.0	77.2	70.3

Abbreviations: JNC7, Seventh Report of the Joint National Committee on Prevention, Detection, Evaluation, and Treatment of High Blood Pressure; JNC8, 2014 Evidence-Based Guideline for the Management of High Blood Pressure in Adults: Report From the Panel Members Appointed to the Eighth Joint National Committee; NQF 0018, National Quality Forum 0018.

a Values are percentages and may not sum to 100% because of rounding or missing data.

b Proposed revisions to JNC7, colloquially known as JNC8, were not sanctioned by any federal or private body at the time of this article.

## Discussion

In the same cohort of patients, estimates of BP control varied up to 16.1 percentage points across different BP thresholds derived from clinical quality metrics and treatment guidelines. Unsurprisingly, the highest proportion of patients categorized as having controlled BP was observed in the proposed revisions to JNC7; this is due to its having the most lenient benchmarks for older patients and the average patient age in the cohort was 58.1 years (4). As expected, JNC7 produced the lowest estimate because of the strict stipulations for patients presenting with diabetes (a comorbidity associated with poor BP control) and removal of the lenient age criteria (3,6). NQF 0018 demonstrated little variation, because it holds blood pressure control requirements constant across all patients.

Because these EMR data come from a large, diverse population, these results are highly generalizable. Furthermore, this analysis was able to use data for patients with pre-existing hypertension, reducing misclassification of patients as nonhypertensive because of failure of diagnosis codes to carry forward into the most recent visit in EMR data.

All BP control algorithms employed in this analysis use thresholds derived from evidence-based guidelines and metrics. Furthermore, these guidelines and metrics serve different, albeit related, purposes. Although the BP thresholds used in treatment guidelines should align with clinical quality improvement metrics, other considerations are important when establishing these algorithms. For example, having computationally simpler quality metrics, such as NQF 0018, to estimate performance reduces the burden of calculating and reporting for clinics that may not have the resources to carry out more advanced analytics. In addition, a simpler metric facilitates better incorporation of standards into clinical practice.

Nonetheless, this analysis demonstrates that careful selection of BP control algorithms is warranted to ensure that 1) research that explores BP control can be validly synthesized into meta-analyses and systematic reviews to inform practice, because even minor differences in algorithms may result in incongruent findings; and 2) BP control estimates across different populations or clinics are comparable and generalizable. Finally, if metrics used for assessing quality do not align with treatment guidelines, guidance should be provided to clinicians on how to appropriately integrate these different algorithms into clinical practice.

A limitation of this analysis is that these results cannot be extended beyond patients with primary hypertension. Regardless, our findings indicate that minor discrepancies in BP control algorithms affect estimates of BP control; thus, mindfulness of these discrepancies is necessary when providing guidance to clinicians, interpreting data, comparing population-wide estimates, and generalizing findings of research.
